# Pharmacogenomic landscape of TNF inhibitors in the Middle Eastern Qatari population

**DOI:** 10.3389/fimmu.2025.1674889

**Published:** 2025-11-19

**Authors:** Zainab Jan, Dinesh Velayutham, QPGx-TNFi Consortium, Borbala Mifsud, Georges Nemer, Puthen Veettil Jithesh, Nady El Hajj, Ehsan Pourkarimi, Omar Albagha, Farah El Assadi, Zainab Jan, Dinesh Velayutham, Samar Al Emadi, Karima Becetti, Mohammed Hammoudeh, Hala Albakheet, Nour Hamad, Sanaa Sharari, Sharon Bout-Tabaku, Buthaina Al Adba, Mona El Chawli, Fathima Abubacker, Mamoun Elawad, Nazira Ibrahim, Borbala Mifsud, Puthen Veettil Jithesh

**Affiliations:** 1 College of Health & Life Sciences, Hamad Bin Khalifa University, Education City, Doha, Qatar; 2 William Harvey Research Institute, Queen Mary University of London, London, United Kingdom; 3 Pharmacology and Therapeutics, Institute of Systems, Molecular and Integrative Biology, University of Liverpool, Liverpool, United Kingdom

**Keywords:** TNF inhibitors, whole genome sequencing, precision medicine, pharmacogenomics, Qatar, autoimmune diseases, drug response

## Abstract

**Introduction:**

Tumor necrosis factor alpha (TNF-α) is an important cytokine that frequently contributes to the pathogenicity of autoimmune diseases. Therefore, TNF inhibitors (TNFi) are used to treat autoimmune diseases. However, around 40% of the patients do not respond to TNFi, with genetic variants being a contributor to this variance. The prevalence of genetic variants affecting TNFi response in Middle Eastern populations is still not understood.

**Methods:**

We assessed the distribution of variants in 111 genes associated with TNFi in 14,387 Qatari individuals using whole genome sequencing data.

**Results:**

Of the 151 known pharmacogenomic variants associated with response to TNFi, approximately half have significantly different allele frequency distribution in the Qatari population compared to other world populations from the gnomAD dataset. High frequency of rs1800629 (*TNF*), rs1800896 (*IL10*), and rs1143634 (*IL1B*) variants are observed, which are known to be associated with responses to Etanercept and Infliximab. Moreover, we identified that *PSORS1C1* has the highest CAP_LoF_ (cumulative allele probability) scores for loss-of-function variants, which is associated with response to Etanercept and Adalimumab.

**Discussion:**

The findings of this study will enhance our understanding of the pharmacogenomics of TNF inhibitors in Qatar and beyond, while also supporting the study of genetics in underrepresented populations.

## Introduction

Autoimmune diseases are the major causes of morbidity and mortality both in the developed and developing countries (1). There are more than 100 different types, such as rheumatoid arthritis (RA), psoriasis, and systemic lupus erythematosus (SLE), affecting approximately 10% of the population globally. Autoimmune diseases are one of the top ten causes of death in women (2). Tumor necrosis factor (TNF) is a key inflammatory molecule whose up-regulation plays a crucial role in the development and pathogenicity of many autoimmune diseases (3). Several *in vitro* and *in vivo* studies demonstrated the dysregulation of TNF-α in autoimmune disease patients (4, 5). Therefore, TNF inhibitors (TNFi) are widely utilized for the treatment of various autoimmune diseases (6, 7). To date, five TNFi, Etanercept (ETN), Adalimumab (ADA), Certolizumab (CZP), Golimumab (GOLI), and Infliximab (IFX) have been approved by the US FDA (8). These inhibitors showed potential activity against TNF-α in patients with above-mentioned diseases, however, treatment response varied substantially, up to 40% of patients showing no positive clinical response (8–10). In rheumatoid arthritis (RA), 30%-40% of patients experience treatment failure with TNFα antagonists, including primary non-response, secondary loss of response, or adverse side effects (11). Furthermore, 23%-46% of inflammatory bowel disease (IBD) patients lose their response to treatment after 12 months (12, 13).

We recently reviewed and summarized the pharmacokinetics, pharmacodynamics, and especially the pharmacogenomics of TNFi, with a particular focus on the influence of HLA and other genetic variants on treatment response and safety profiles (14). A previous study conducted on IBD patients reported that who carried HLA-DQA1*05 showed highest rate of immunogenicity when treated with Infliximab and Adalimumab (15). Another study conducted on IBD patients reported that HLADQA1*05A>G variant is associated with a significantly higher risk of infliximab antibody formation and loss of response (16). Furthermore, previous genome-wide association studies (GWAS) (17) explored the genetic variants associated with response to TNFi. A previous study on the Italian population reported a significant association between the TNFα -308 (rs1800629) polymorphism and Behçet syndrome susceptibility. Moreover, they reported that the GA genotype was found at a higher frequency in patients compared to healthy controls (18). Similarly, in 74 Behçet syndrome patients treated with anti-TNFα therapy, they found that the GA genotype was more frequent among non-responders, while the GG genotype predominated in responders, suggesting a possible role of rs1800629 as a predictive biomarker of treatment response (19).

In the Middle East region, the severity of RA is comparable to other global regions, with 12% of patients reporting low disease activity (20). In Qatar, more than 2000 patients are treated for RA every month. The disease itself also has a strong genetic component, ~60% of RA disease variability in Qatar was shown to be inherited, and two novel risk loci were identified in addition to the known ones (21). Moreover, epidemiological studies from Qatar reported a significant increase in rheumatic manifestations in IBD patients compared to the rest of the world, suggesting a common link with RA (22). Data from the RA registry at Hamad Medical Corporation in Qatar shows variability in disease activity scores, with remission rates ranging from 17.5% to 30.3% depending on the scoring method used, highlighting that many patients do not achieve remission despite various treatment regimens, including TNFα inhibitors (23). Numerous studies have identified genetic loci and gene expression patterns linked to TNFi response, with 25 single nucleotide polymorphisms (SNPs) in 5 genes associated with TNFi response in RA, and additional SNPs identified through meta-analysis (24). Since geographical and population differences affect variant distributions, we hypothesize that the distribution of genetic variants affecting response to TNFi may differ in the Middle Eastern population from other world populations. Furthermore, there may be population-specific novel variants in the genes associated with TNFi response in the Qatari population. Here in this study, we used computational approaches to explore the distribution of pharmacogenomic variants associated with TNFi response in the Qatari population.

## Results

### Summary of pharmacogenomic variants

A total of 141,735,839 variants were identified in 14,387 Qatari individuals. A total of 111 TNF-response genes, previously associated with response to TNF inhibitors (TNFi) in various populations were analyzed, and we identified a total of 203,768 variants across these genes in the Qatari population (**Supplementary Figures S1**, **S2**; **Supplementary Tables S1**-**S3**). However, the association of these variants with TNFi response, specifically in the Qatari population, remains to be determined. Notably, 4261 variants in TNF-response genes were classified as having high or moderate impact, while 3757 variants were missense, with 30% of these categorized as singleton variants. The highest number of missense variants were found in the genes *REV3L* (181, 4.8%), *CR1* (168, 4.47%), and *IVL* (105, 2.67%). Moreover, half of the missense variants were rare, with an allele frequency < 0.01. We also identified loss of function (LoF) variants in genes associated with response to TNFi; 38 LoF variants were present in 13 TNFi pharmacogenes (**Supplementary Figure S3**). *CST5* (9 variants) and *IVL* (8 variants) had the highest number of LoF variants (**Supplementary Table S4**). rs1800896 (*IL10*) and rs1143634 (*IL1B*) have been associated with rheumatoid arthritis and inflammatory bowel disease. Moreover, we identified two novel LoF variants in two genes, *GBP6* (chr1:89384209, p. Gln529*, allele frequency: 0.0000341) and *LY96* (chr8:73991522, allele frequency: 0.0000341), in the Qatari population.

### Allele frequency of known pharmacogenomic variants associated with response to TNF inhibitors

**Figure 1:**
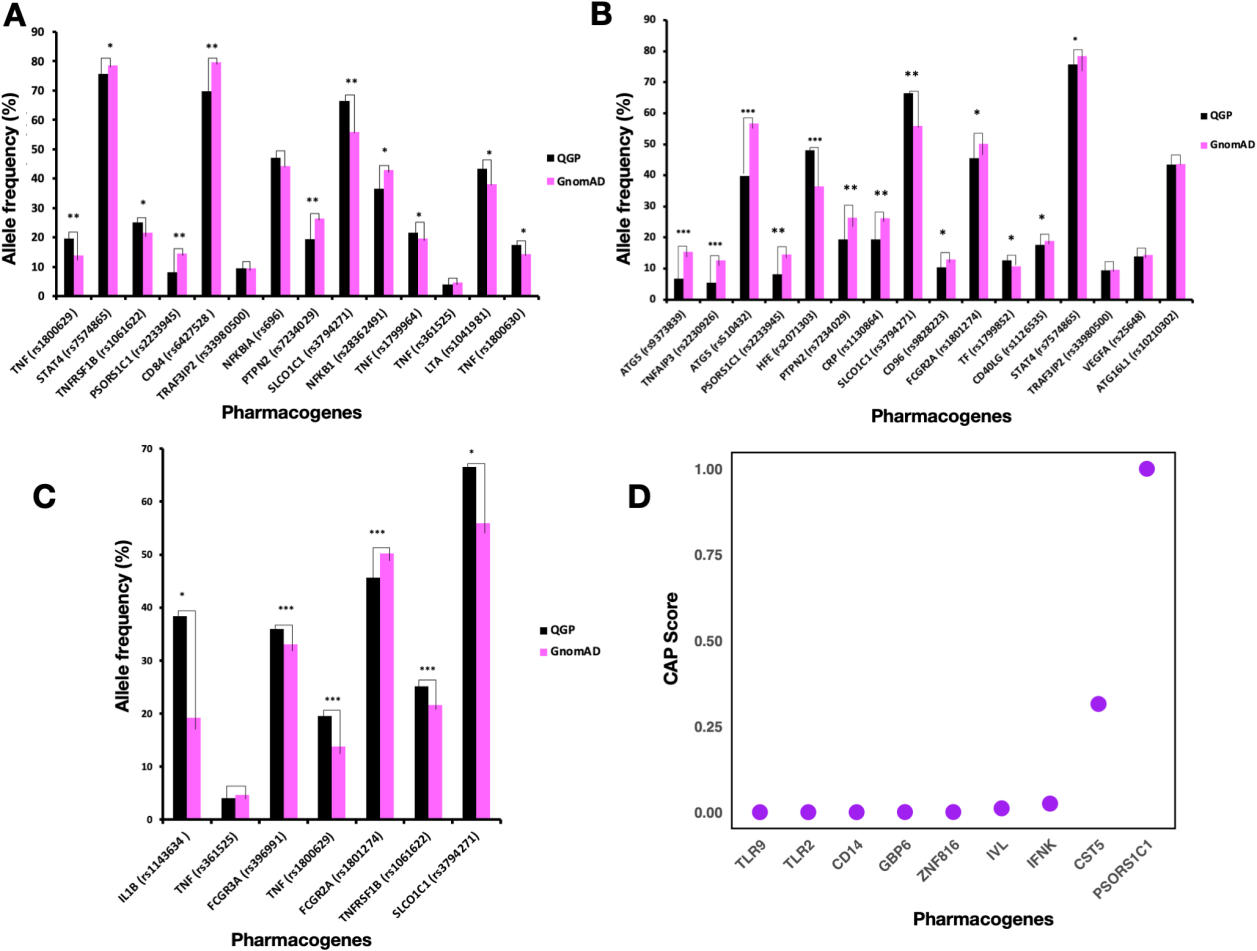
Comparison of Allele Frequencies of Pharmacogenes Associated with Response to Adalimumab, Etanercept, and Infliximab in the Qatari Population. **(A-C)** Comparison of Allele frequencies of pharmacogenes associated with response to Etanercept **(A)**, Adalimumab **(B)**, and Infliximab **(C)**. *P*-values of significantly differing frequencies with Bonferroni adjustment indicated as follows: *P* < 0.05 (*); 10^−49^ ≤ *P* < 10^−20^ (**); *P* ≤ 10^−50^ (***). One asterisk (*) means statistically significant (*P* < 0.05); Two asterisks (**) mean highly significant (*P* between 10^−20^ and 10^−49^). Three asterisks (***) mean extremely significant (*P* ≤ 10^−50^). **(D)** Cumulative Allele Probability score for LoF variants in pharmacogenes associated with TNF inhibitor response.

Diverse distribution of allele and genotype frequencies were observed in the Qatari population compared to other world populations (**Table 1**). Frequencies of 151 variants from 111 genes were analyzed that were annotated in the PharmGKB as related to response to Etanercept (**Figure 1A**), Adalimumab (**Figure 1B**), and Infliximab (**Figure 1C**), as well as multiple TNFi (**Supplementary Tables S5**-**S10**).

**Table 1:** Distribution of variants associated with response to multiple TNF inhibitor in the Qatari population compared to gnomAD, highlighting variants with significant differences in allele frequency.

Gene	Chromosome	Locus	rs ID	Nucleotide change	QGP-AF	gnomAD AF	Variants type
*C9orf72*	Chr9	27543283	rs3849942	c.-4953A>G	0.666701	0.7818	Downstream gene variant
*RSRP1*	Chr1	25243590	rs1043879	c.716A>G	0.315504	0.1868	Missense variant
*MAP3K1*	Chr5	56900777	rs96844	c.*7097G>A	0.722562	0.5875	Downstream gene variant
*IFNGR2*	Chr21	33403138	rs8126756	c.-406T>C	0.136016	0.2437	5 prime UTR variant
*ATXN2L*	Chr16	28826194	rs8049439	n.-3584T>C	0.212344	0.4040	Upstream gene variant
*MAP3K14*	Chr17	45290287	rs7222094	c.256 + 203A>G	0.66574	0.5446	Intronic variant
*CD84*	Chr1	160546518	rs6427528	c.*1738T>C	0.697644	0.796	3 prime UTR variant
*IL1RN*	Chr2	113116890	rs4251961	c.-1129T>C	0.507298	0.295	Upstream gene variant
*TNFRSF1B*	Chr1	12207235	rs3397	c.*215C>T	0.648189	0.5096	3 prime UTR variant
*CTLA4*	Chr2	203874196	rs3087243	c.*1384G>A	0.552096	0.37	Downstream gene variant
*REV3L*	Chr6	111352511	rs240993	n.-778A>G	0.714603	0.54	Upstream gene variant
*IFNG*	Chr12	68161231	rs2069705	c.-1616C>T	0.784493	0.5953	Upstream gene variant
*CNTN5*	Chr11	100140279	rs1813443	c.1581-50847G>C	0.376634	0.2316	Intronic variant
*NLRP3*	Chr1	247448734	rs10754558	c.*230G>C	0.475429	0.6358	Intronic variant
*TNFRSF1B*	Chr1	12208442	rs1061631	c.*1422G>A	0.265031	0.1533	3 prime UTR variant
*ADAM17*	Chr2	9522691	rs10929587	n.*4810A>T	0.755286	0.6351	Downstream gene variant
*FBXL19*	Chr16	30931304	rs10782001	c.1034 + 720G>A	0.677	0.5339	Intronic variant
*IL23R*	Chr1	67222666	rs10489629	c.955 + 2936T>C	0.322062	0.4759	Intronic variant
*TAP1*	Chr6	32847198	rs1135216	c.2090A>G	0.230173	0.1749	Missense variant
*CTNNA2*	Chr2	79673969	rs11126740	c.102 + 22311A>G	0.763573	0.627	Downstream gene variant

The rs1800629 variant in the *TNF* gene had a higher allele frequency (around 19.5%) in the Qatari population as compared to other world populations (*P*-value = 1.89 × 10^-21^). This variant is associated with response to Etanercept and belongs to Level 2B in PharmGKB annotations. On the other hand, patients with the rs1800896 (*IL10* gene) variant showed a positive response to Etanercept in rheumatoid arthritis (25), which means the high prevalence of GG genotype in the Qatari population points towards a probable better response to Etanercept as compared to other populations. The rs1041981 variant in the *LTA* gene had allele A in around 43% of the Qatari individuals studied. In comparison, the distribution of this allele was slightly higher in the African population, approximately 50% (*P*-value = 6.21 × 10^-12^). This allele was associated with a better response to Etanercept in patients with Rheumatoid Arthritis (26). On the other hand, for the rs1800630 in *TNF*, the Qatari population had a higher frequency of allele A (17.46%) as compared to the gnomAD (14.43%) (*P*-value = 2.34 × 10^-7^). This allele was associated with better response to Etanercept in patients with Rheumatoid Arthritis (26). Moreover, the Qatari population with *3/*3 diplotype of *CYP3A5* accounted for 33.1%. Previous studies found that this diplotype was associated with increased response to Etanercept (27). For the variant rs3794271 in the *SLCO1C1* gene, the allele frequency in the Qatari cohort (66.5%) was higher than in the gnomAD dataset (55.9%) (*P*-value = 3.95 × 10^-47^). This allele was associated with a decreased response to Etanercept in patients with Rheumatoid Arthritis (28). The rs1800471 (*TGFB1*) variant was 6.7% in the Qatari population, while the AF was around 8% in the non-Finnish European population. Studies showed that allele C in rheumatoid arthritis patients was associated with non-responsiveness to Etanercept (25), thus Qatari patients with this allele could have no response to this TNFi.

Some rare variants in the *TNF* gene related to Infliximab response identified in the East Asian, American & non-Finnish European populations were not present in the Qatari population, including rs4987086, rs3093548, rs2736195, rs55634887, rs55994001, and rs4248163. The frequency of rs1143634 variant in *IL1B* was 38.3% in the Qatari population in comparison to 19.24% in gnomAD. Allele G of this variant was associated with decreased response to Infliximab in Crohn’s disease (CD) patients (29). Hence the higher frequency of this allele in the Qatari population suggests more people may be at risk of poor response when treated with Infliximab. In addition, for *TNFRSF1B* (rs1061622), an important member of the TNF superfamily, Qataris had a higher prevalence of allele G, around 25%, (*P*-value = 2.35 × 10^-7^). Allele G was associated with a worse response to TNF blockers in people with rheumatoid arthritis (25). The allele frequency of rs2476601 (*PTPN22*), rs352139 (*TLR9*), rs1800630 (*TNF*), and rs1813443 (*CNTN5*) that are associated with response to multiple TNF inhibitors were higher in the Qatari population as compared to all the other world populations from the gnomAD datasets. However, the allele frequency of variants rs396991 (*FCGR3A*), rs4810485 (*CD40*), and rs1120902 (*IL23R*) was very low in the Qatari population as compared to other world populations.

The frequency of allele C in the rs9304742 (*ZNF816*) variant was 44% in the Qatari population, lower than African, Amish, South Asian, and Jewish populations. At the same time, it is higher than other populations, including Finnish European, non-European, East Asian, and American populations. Studies identified that the carriers of allele C had decreased response to Etanercept and other TNF-alpha blockers such as Adalimumab and Infliximab (30). The prevalence of the G allele in *TNFRSF1B* (rs1061622) was highest in the Qataris, followed by South Asians. Previous studies reported that the rs1061622 variant increased the risk of adverse effects in rheumatoid arthritis upon anti-TNF treatment (31). Qataris had a high prevalence of this allele, suggesting the potential for having a lower response and side effects for patients during anti-TNF therapy.

For Adalimumab, the *TF* gene displayed the highest number of genetic variants in the Qatari population, followed by *TNFAIP3*. The functional variants in the *TF* gene had a score of 0.0045 variants per participant, while *TNFAIP3* had a score of 0.0032, and *ATG5* had the lowest score at 0.0002 (**Supplementary Figure S4**, **Supplementary Tables S11**, **S12**). In the case of Etanercept, the *TNFRSF1B* gene also had the highest number of variants, followed closely by *PTPN2*, with functional variant scores of 0.003 for *TNFRSF1B* and 0.0029 for *PTPN2*, while *TNF* had a score of 0.00069 (**Supplementary Figure S5**, **Supplementary Tables S11**, **S12**). Similarly, for Infliximab, *TNFRSF1B* again showed the highest number of variants, followed by *FCGR2A* and *FCGR3A*, with scores of 0.003, 0.0028, and 0.0023 variants per participant, respectively (**Supplementary Figure S6**, **Supplementary Tables S11**, **S12**).

### Linkage disequilibrium analysis of the important pharmacogenomic variants

The Linkage Disequilibrium (LD) analysis revealed varying degrees of allele associations across different SNP pairs associated with response to TNF inhibitors (**Supplementary Table S13**). We found that the rs909253 variant on the *TNF* gene was in very strong LD with rs1041981 (r² = 0.9932). rs1041981, located in the *LTA* gene, has been associated with response to Etanercept (26), while rs909253 is associated with multiple TNF inhibitors (32). Similarly, a moderate LD was observed between rs1799964 and rs1800630, with an r² value of 0.7619, suggesting a moderate association between these loci. Both variants are located on the *TNF* gene and are associated with response to Etanercept (26). On chromosome 9, several SNP pairs exhibited strong to moderate LD. The strongest LD was observed between rs868856 and rs7046653 (r² = 0.9902), followed by rs3849942 and rs774359 (r² = 0.8499). rs868856 and rs7046653 are located on the *MOB3B* gene, while rs3849942 and rs774359 are located on *C9orf72*. All four variants are associated with response to multiple TNF inhibitors (33). The SNP pair rs868856 and rs774359 showed a moderate association (r² = 0.7155), while rs868856 and rs3849942 demonstrated a slightly weaker but still significant LD (r² = 0.6887). Notably, rs7046653 and rs3849942 also exhibited moderate LD (r² = 0.6855). On chromosome 7, the pair rs854548 and rs854555 showed a relatively weaker LD (r² = 0.4676), while the SNP pair rs1800750 and rs361525 on the *TNF* gene showed the lowest LD in this analysis (r² = 0.3522), indicating a weak association. rs854548 are rs854555 are located on *PON1* and both are associated with response to multiple TNF inhibitors (33). rs361525 is associated with response to Etanercept (34), while rs1800750 showed no association with response to Infliximab (35). Overall, these results suggest that certain SNP pairs, particularly on chromosomes 6 and 9, exhibit strong LD, which could have significant implications for understanding genetic factors influencing TNF inhibitor (TNFi) response. The high LD between these variants may reflect shared genetic pathways that contribute to treatment efficacy, adverse effects, or both, offering potential markers for predicting patient-specific responses to TNFi therapy.

### Pharmacogenetic risk profile

To assess the potential risk associated with genetic variants in a population, we calculated the cumulative allele probability (CAP). This score captures both the number of functional variants and their allele frequencies within a gene, representing the probability that individuals in the population carry at least one variant allele in a given gene. We calculated the CAP score for all the missense variants (3757), and LoF variants (**Figure 1D**) separately (**Supplementary Tables S14**, **S15**). The genes associated with Infliximab response with the highest CAP scores for missense variants included *FCGR3A* (0.021) and *FCGR2A* (0.01). The genes associated with Adalimumab response with the highest CAP score for missense variants included *TF* (0.048) and *TRAF3IP2* (0.02). Etanercept-associated genes with a comparable CAP score for missense variants included *PSORS1C1* (0.03) and *TRAF3IP2* (0.02). In the case of all the missense variants, *CR1* (0.178*)* and *RHD* (0.108) had the highest CAP scores that are associated with response to multiple TNFi. The genes with the highest CAP_LoF_ scores were associated with response to multiple TNFi, including *PSORS1C1* and *CST5*.

### Genotype frequency distribution of pharmacogenomic variants known to be associated with response to TNF inhibitors

**Figure 2:**
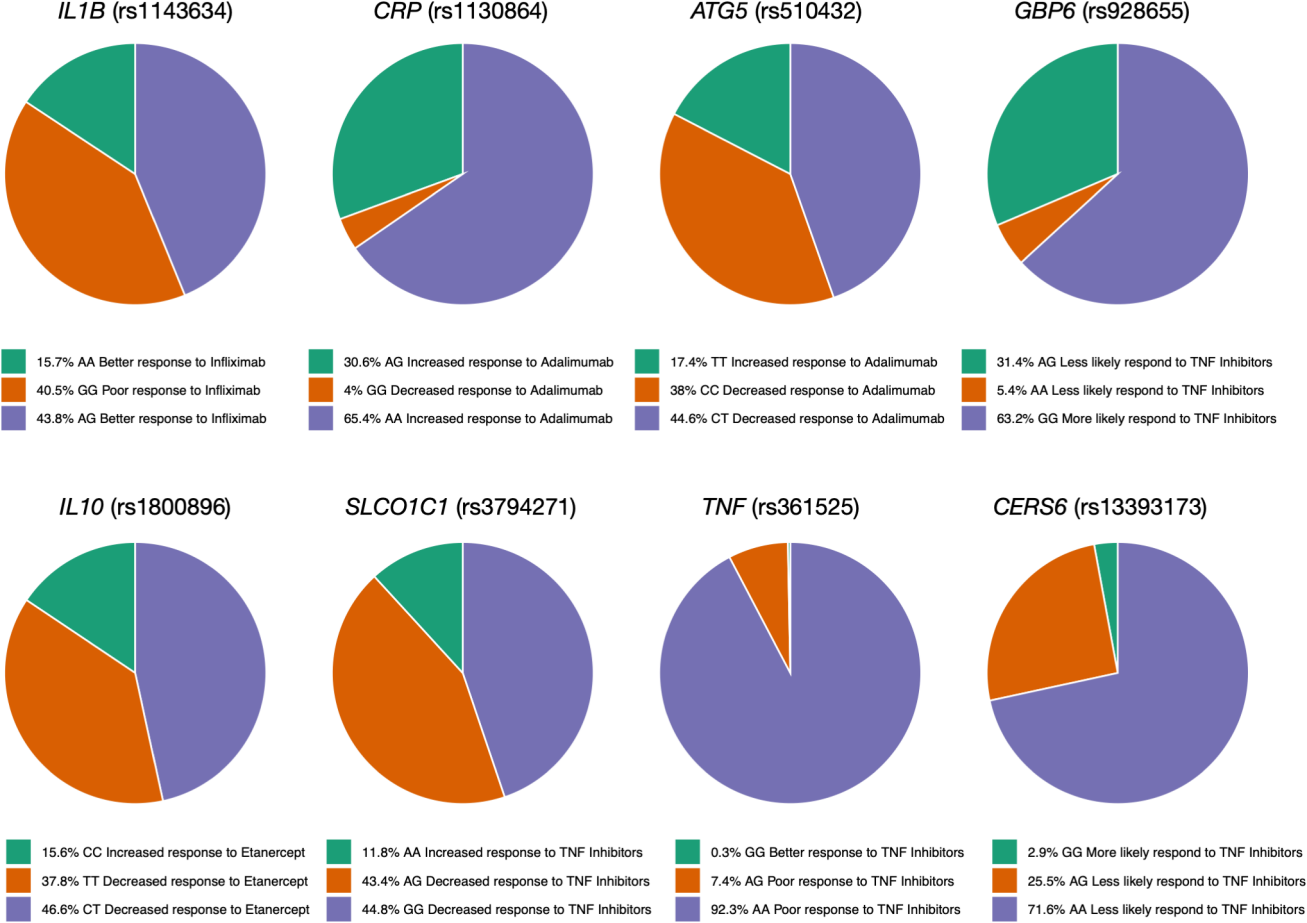
Genotype frequency of some variants associated with TNF inhibitors in the Qatari population. Key variants include *IL1B* (rs1143634), which is more frequent in Qataris (~40.5%) and linked to lower Infliximab response, and *TNF* (rs4248159). The *CRP* (AA genotype) is linked to increased Adalimumab response.

We also calculated the genotype frequency of variants associated with TNFi response in the Qatari population. In the case of *IL1B* (rs1143634), the Qatari population had the GG genotype frequency of 40.5%, which was associated with lower response to Infliximab in Crohn’s disease patients (35) (**Figure 2**). The genotype frequencies (AA and AC) of rs2431697 in *PTTG1* were 81% in the Qatari population; studies showed that these genotypes were associated with a decreased response to Infliximab in Crohn’s disease patients. The genotype CC and CT in *HFE* associated with response to Adalimumab in Crohn’s Disease patients were associated with a decreased likelihood of response to Etanercept in people with Rheumatoid Arthritis (36). Moreover, the genotype frequency of AA in the *CRP* gene is 65.4% in the Qatari population, which has been associated with an increased response to Adalimumab (**Figure 2**).

### Functional consequences of rare variants in *TNF*

**Figure 3:**
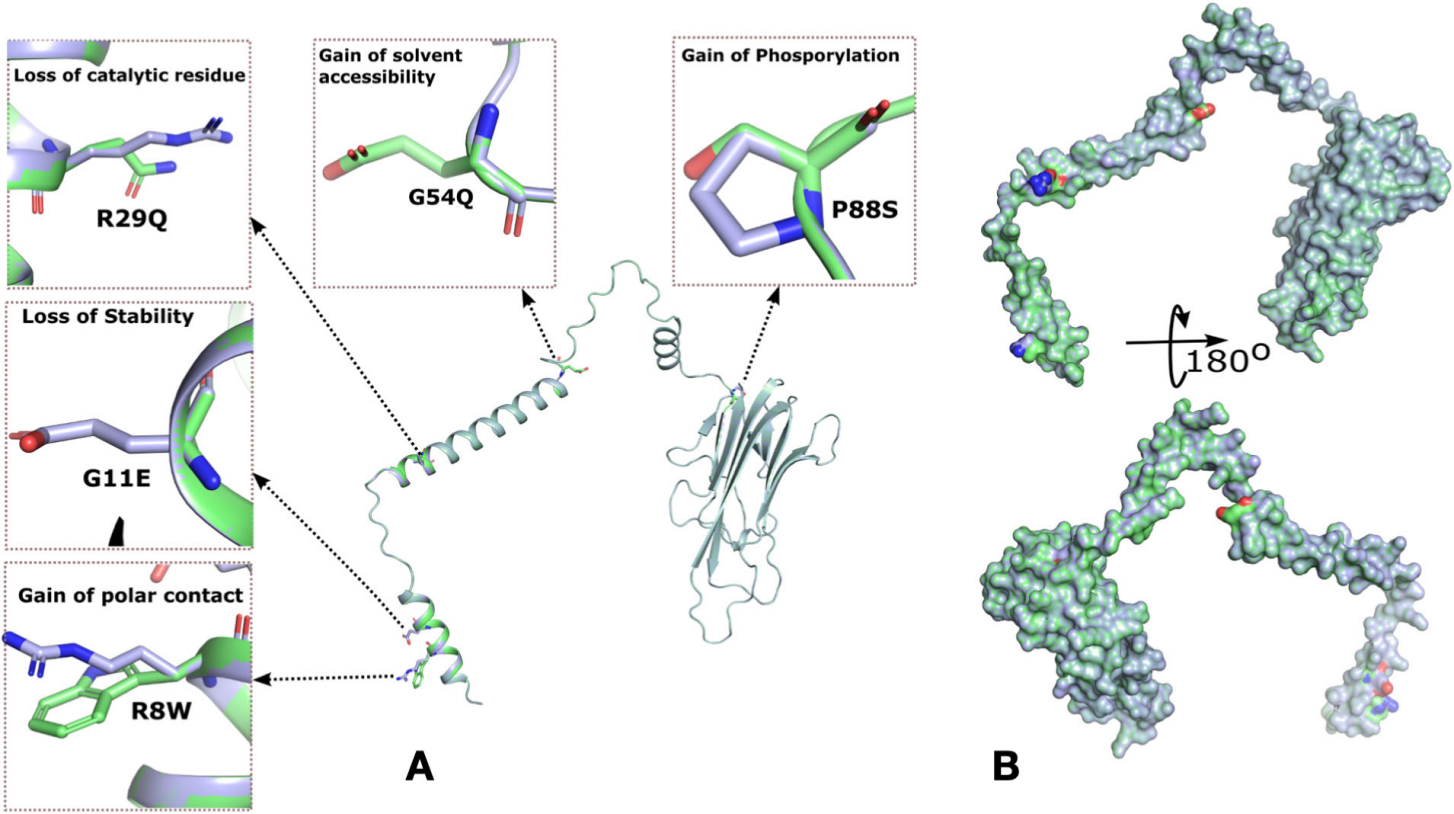
Rare mutations in TNF-α identified in the Qatari population and molecular docking analysis of wild-type and mutant TNF-α with TNFR1. **(A)** Surface representation of TNF-α highlighting five missense mutations and their predicted effects on protein structure and function. The hydrophobic surface around the binding site is also shown. **(B)** Cartoon representation of TNF-α, depicting overall structural organization and the location of the identified mutations.

We identified 10 rare missense variants in *TNF* gene in the Qatari population, out of which 5 variants had a CADD score greater than 20 (**Tables 2**, **S16**). MutPred, a machine learning tool was used for the prediction of the effect of mutations on the protein function (**Figures 3A, B**). The p. Glu11Gly mutation in TNF-α causes the loss of disorder (Predicted conservation scores PCS = 0.0606), loss of stability (PCS = 0.0821), whereas the mutation p. Ala16Val was responsible for loss of helix (PCS = 0.0196), gain of loop (PCS = 0.0312), loss of phosphorylation at Ser27 (PCS = 0.0818). Furthermore, the variant p. Gly54Glu causes the gain of sheet (PCS = 0.0344), loss of helix (PCS = 0.0558), gain of solvent accessibility (PCS = 0.0837), gain of loop (PCS = 0.0851) while p. Pro88Ser causes the gain of phosphorylation at Pro88 (PCS = 0.0475), gain of MoRF (motif recognition factor) binding (PCS = 0.0728), gain of glycosylation at Pro88 (PCS = 0.095). The p. Arg107His associated with the loss of MoRF binding (PCS = 0.0142). Moreover, we observed that the p. Thr181Asn variant on TNF-α is responsible for the loss of phosphorylation at Thr181 (PCS = 0.0043), loss of disorder (PCS = 0.0587), gain of helix (PCS = 0.0854), loss of loop (PCS = 0.0986), although the CADD score of this variant was lower than 20. dbNSFP v4.2a, a hub of 37 machine learning algorithms, was used to annotate these rare variants (**Table 2**). Functional annotation from six tools is presented in **Table 2**. rs14054183, rs374531985 were predicted as deleterious, rs758704433, rs57662166, rs5485326242 as probably damaging, whereas other variants were classified as benign, neutral, and tolerated. The variants p. Glu11Gly and p. Pro88Ser are highly deleterious missense variants in the Qatari population.

**Table 2:** Rare and novel missense variants in *TNF* gene present in the Qatari population and their predicted functional consequences.

ID	Position in chr 6	REF	ALT	AF	cDNA	Protein name	CADD score	M-CAP	MetaLR	PolyPhen-2	FATHMM	Mutation taster	SIFT	Final classification
rs3745319	31575763	C	T	0.000443111	c.22C>T	p. Arg8Trp	25.7	Deleterious	Deleterious	Deleterious	Deleterious	Deleterious	Tolerated	Deleterious
rs763000109	31575788	C	T	0.000068171	c.47C>T	p. Ala16Val	1.002	Benign	Benign	Benign	Benign	Benign	Deleterious	Benign
rs576621666	31575827	G	A	0.000102256	c.86G>A	p. Arg29Gln	22.7	Deleterious	Deleterious	Deleterious	Deleterious	Deleterious	Tolerated	Deleterious
rs756182468	31575902	G	A	0.000170427	c.161G>A	p. Gly54Glu	22.1	Deleterious	Deleterious	Benign	Deleterious	Deleterious	Tolerated	Deleterious
rs4645843	31576785	C	T	0.00538551	c.251C>T	p. Pro84Leu	14.32	Deleterious	Deleterious	Deleterious	Deleterious	Deleterious	Tolerated	Deleterious
rs75870443	31577155	G	A	0.000340885	c.320G>A	p. Arg107His	8.579	Benign	Deleterious	Deleterious	Benign	Benign	Tolerated	Benign
rs548532642	31577184	G	A	0.000340885	c.349G>A	p. Val117Met	3.939	Benign	Deleterious	Deleterious	Benign	Benign	Tolerated	Benign
rs140654183	31577377	C	A, T	0	c.542C>A	p. Thr181Asn	17.51, 14.80	Deleterious	Deleterious	Deleterious	Deleterious	Deleterious	Tolerated	Deleterious
.	31575773	A	G	0.000068171	c.32A>G	p. Glu11Gly	32	Deleterious	Deleterious	Deleterious	Deleterious	Deleterious	Deleterious	Deleterious
.	31576796	C	T	0.000340885	c.262C>T	p. Pro88Ser	24.7	Deleterious	Deleterious	Deleterious	Deleterious	Deleterious	Deleterious	Deleterious

Final classification of each variant is based on integrating the evidence from the different functional prediction tools.

### Interaction of the mutant and wild type TNF with its receptor protein TNFR1

**Figure 4:**
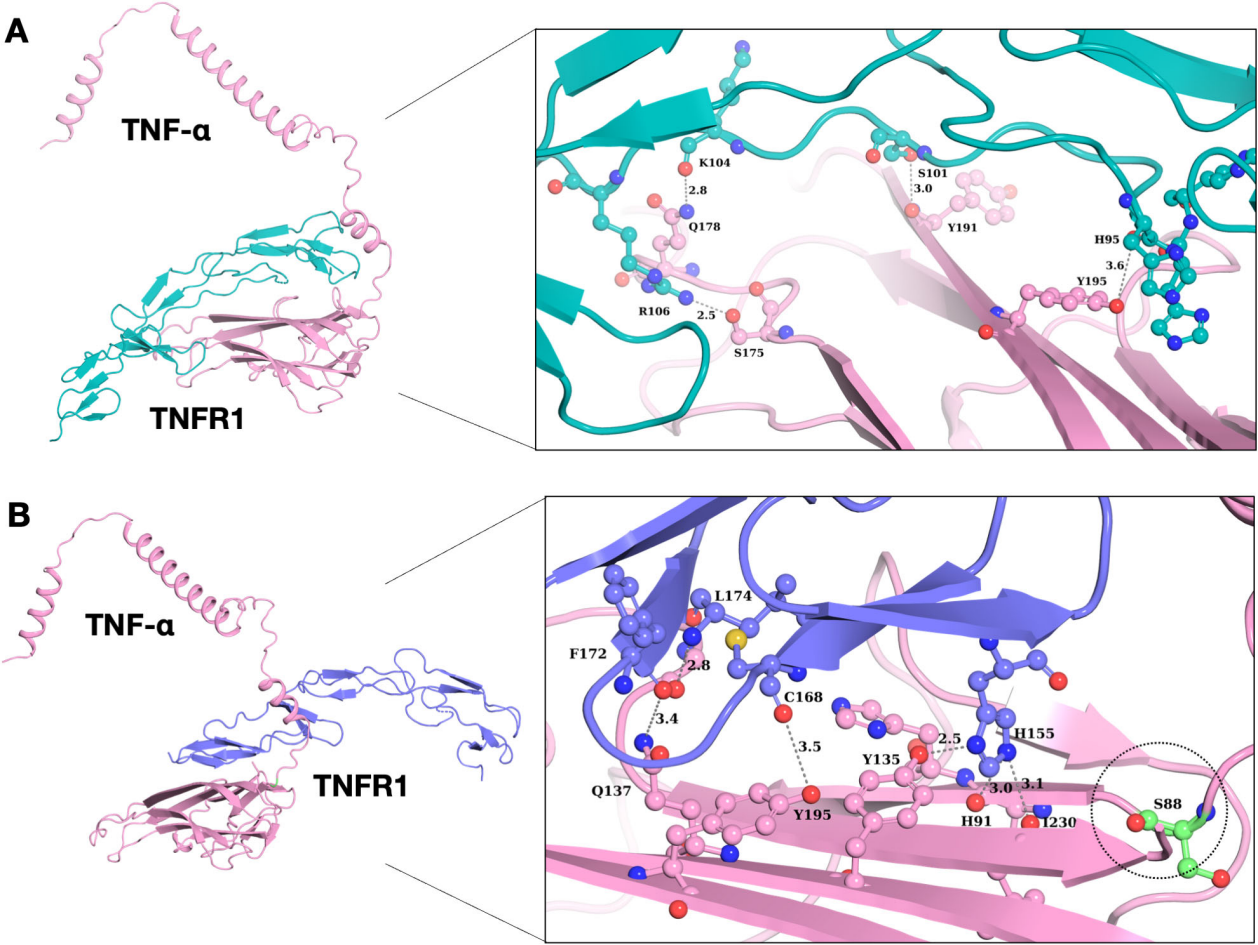
Interaction analysis of wild type and mutant TNF-α with TNFR1. **(A)** Binding pattern of wild type TNF-α with TNFR1, demonstrating structural alterations of wild type TNF-α with its receptor protein. **(B)** Binding pattern of mutant TNF-α (P88S) with TNFR1. The P88S mutation enhanced hydrogen bonding propensities in neighboring residues, potentially affecting TNF-α/TNFR1 interactions.

Previous studies have reported that TNF binds to TNFR1, leading to the activation of the signaling pathway. TNF inhibitors block the TNF-TNFR1 interaction. Here in this study, we identified two novel and three rare missense variants related to the *TNF* gene, but only one mutation was localized in the binding region of TNF (Pro88Ser). We performed the docking of both the wild type and mutated TNF with TNFR1. Both mutant and wildtype are TNF-α shown in pink color (**Figures 4A, B**). Out of the five variants, only one variant increased the binding affinity of TNF with TNFR1. The P88S variant confers a significant increase in the binding affinity of the mutant TNF with TNFR1. The wild type of TNF forms four hydrogen bonds with binding affinity of -266.34 kcal/mol, whereas the binding affinity of the mutated TNF (Pro88Ser) with wild type TNFR1 was -280.30 kcal/mol (**Figures 4A, B**).

## Discussion

We conducted an extensive survey of pharmacogenomic variation associated with TNF inhibitor response in the Qatari population. The observed differences in the allele frequency distributions highlight the unique genetic makeup of the Qatari population and emphasize the importance of tailoring therapeutic approaches to local genetic characteristics. Moreover, it emphasized the significance of rare genetic variations in influencing pharmacogenes’ protein function.

Our analysis revealed that a lot of variants are rare and singletons in TNFi response-associated pharmacogenes, with deleterious variants more likely to be rare (37). This trend was supported by the high prevalence of rare missense variants in the examined pharmacogenes, consistent with prior research on drug target genes (38, 39). Additionally, low-frequency functional variants, which are predominantly rare, are often not sufficiently addressed by conventional genotyping arrays (40). Pharmacogenes with the highest number of missense variants in the Qatari population was *REV3L* and *CR1*. Moreover, we found that the *CR1* (0.0125 variants per participant) gene has the highest number of functional genetic variants in the Qatari population. A previous study investigated genetic variants affecting erythrocyte sedimentation rate (ESR) in patients with severe active rheumatoid arthritis (RA) and found that the *CR1* rs6691117 genotype showed a significant association with baseline ESR levels (*P* = 0.01) (41). Another study found the presence of two copies of the AA major allele at rs6691117 in the *CR1* gene is linked to reduced baseline ESR levels before anti-TNF therapy (42). *CR1* acts as a potent inhibitor of complement activation, and genetic variations within this gene could influence Rouleaux formation, consequently affecting ESR levels. The rs6691117 SNP introduces a non-synonymous change from isoleucine to valine, possibly altering the secondary structure of CR1. This alteration might impact the ability of CR1 to clear complement opsonized immune complexes, potentially leading to increased ESR levels (41).

The prevalence of a high number of functional variants in *CR1* underscores the potential clinical relevance in guiding treatment decisions. These findings offer valuable insights for clinicians, enabling them to better predict patient responses to TNF inhibitors and optimize treatment strategies accordingly. Moreover, the study’s approach of assessing both known and novel variants across a wide range of genes associated with TNF inhibitors provides a comprehensive understanding of the genetic landscape influencing treatment response. This holistic approach enhances the accuracy of predictive models, enabling more precise identification of individuals at risk of treatment non-response or adverse reactions. Furthermore, we identified that *CR1* with the highest cumulative allele probability score (CAP) associated with multi-TNF inhibitor response highlights potential targets for further investigation and therapeutic intervention. Understanding the genetic factors influencing response to specific treatments can inform the development of personalized medicine approaches tailored to individual patient profiles (43).

This study has several limitations. There is a lack of experimental data related to the association between drug responses for the novel pharmacogenomic variants identified. This study lacks data on responders and non-responders to TNF blockers. So, it was not possible to predict the association of the novel variant with drug response. We predicted the phenotypic consequences of missense variants associated with TNFi response-related genes, which is a crucial aspect of precision medicine as it helps in translating the genomic data into clinically actionable information. Functional interpretation of novel variants relies on computational tools that predict phenotypic effects based on structural features, sequence homology, and evolutionary constraints (44). However, we used several machine learning tools including meta-predictor CADD for functional prediction. Jagadeesh et al. (45) reported that many variant classifier tools misclassify pathogenic variants as benign, including SIFT (38%), CADD (26%), POLYPHEN-2 (31%), and MetaLR (27%). So, further studies are required for clinicians to use the prediction and classification of novel pharmacogenomics variants based on these tools. However, in this study, we also used the M-CAP tool that classifies pathogenic and benign variants with 95% accuracy (45). Further studies are required to generate more evidence for the association of the variants studied and clinical implementation for predicting TNFi response accurately. Our study provides valuable insights into the pharmacogenomic variants associated with TNF inhibitor response; however, it does not incorporate functional data such as protein abundance, enzyme/transporter activity, or drug concentration in target tissues. These non-genetic factors, though critical to precision dosing, remain underexplored and represent an important avenue for future research (46).

Our study unveiled the distribution of pharmacogenomic variants affecting response to anti-TNF treatment in the Qatari population. We observed a distinct allele frequency distribution in Qatar compared to other populations. Integrating genetic insights into clinical practice can enhance treatment efficacy, minimize adverse reactions, and ultimately improve patient outcomes in the management of autoimmune diseases and other conditions requiring TNF inhibitor therapy. Further clinical studies are required to enhance the evidence for use of these pharmacogenes in personalized gene-based medication prescriptions.

## Materials and methods

### Study design and data collection

The study follows a systematic workflow to investigate the pharmacogenomics of TNF inhibitors in the Qatari population using WGS data generated from an observational longitudinal cohort as described in the next sections. First, we curated pharmacogenetic variants related to TNF inhibitor response from PharmGKB. These variants were then mapped onto the Qatari WGS dataset to determine their distribution and prevalence within the local population. To further interpret their functional and clinical relevance, the identified variants underwent annotation using bioinformatics tools to assess their potential impact on gene function and drug response. Next, linkage disequilibrium (LD) analysis was performed to evaluate the co-inheritance of these pharmacogenetic variants with other genetic variants. Additionally, we compared the allele frequencies of these variants in the Qatari population with those reported in global GnomAD to identify population-specific pharmacogenetic differences. Finally, we computed a cumulative risk probability score, integrating multiple pharmacogenetic variants to estimate the likelihood of altered TNF inhibitor response in the Qatari population. The complete workflow of the study is illustrated in **Supplementary Figure S1**.

### Ethical approval and the study population

Ethical approval for this study was obtained from the Qatar Biobank (QBB) institutional review board (IRB) under the protocol number QF-QBB-RES-ACC-016 as a retrospective data analysis study. The study population comprised 14,387 Qatari individuals previously recruited by the QBB, where the participants provided written informed consent to participate. The genomes of these individuals were sequenced as part of the first and second phases of the Qatar Genome Program (QGP). The cohort includes 8026 women and 6361 men, and the mean age of the included individuals was 41.1 (SD 13.1). The study included Qatari and long-term residents (≥15 years living in Qatar) aged 18 years and above.

### Whole genome sequencing and data processing

The sequencing and data processing were done as described previously and briefly described here (47, 48). The genomic DNA was isolated from blood using standard procedures adopted from QIASymphony. Illumina TruSeq DNA Nano kit standard procedure was adopted for genomic DNA library preparations from 150 ng of total DNA and sequenced using HiSeq X Ten to have at least a 30× mean coverage. FASTQ files were assessed using FastQC (v0.11.2) (https://www.bioinformatics.babraham.ac.uk/projects/fastqc/). Raw reads were mapped to human reference genome assembly GRCh38 using bwaKit (v0.7.12) (49). Variant calling was carried out by using Sentieon tool (50), and a variant call file was generated by employing Haplotyper.

### Variant identification

All the genes and variants associated with TNFi response were collected from two resources: 1) PharmGKB (51) comprising clinical variant data (downloaded in December 2023); 2) TNFi response-related pharmacogenomics scientific publication (27, 52). From PharmGKB, we retrieved 391 genes and 400 variants that were known to be associated with response to TNFi, out of which 3 genes were collected from recent scientific publications (27, 52). Among these, 118 genes with 161 variants remained after removing the duplicate data for same genes and variants collected from multiple sources. In the second phase of screening, we found 10 HLA haplotype variants in 7 HLA genes and removed them. Finally, 111 genes with 151 variants were selected, that also include *CYP2C9*, *CYP2D6*, and *CYP3A5*. The variants selected above were extracted from the QGP WGS data using BCFtools (53).

### Variant annotations and linkage disequilibrium

gnomAD v3.1.2 (54), ALFA (55), and dbSNP (56) databases were used for the annotation of known and novel variants associated with TNFi response. Tabix was employed to extract all variants from the input vcf file. SnpEff/SnpSift v4.3 were used for the annotation of variants, which categorized variants as high-, low-, modifier-, or moderate-impact variants based on their possible impact on the protein (57). Moreover, we used LOFTEE to extract the loss of function variants (58). The high impact category included loss-of-function (LoF) variants. For the star allele calling in *CYP2C9*, *CYP2D6*, and *CYP3A5*, Aldy4 was employed (59). Moreover, we used PLINK for linkage disequilibrium analysis (60).

### Statistical analysis

We computed the allele and genotype frequencies of the known variants in the Qatari population directly from the extracted VCF file. To assess the differential distribution of allele frequencies between the Qatari population and gnomAD datasets, we conducted Chi-square tests, Fisher’s exact tests, and two-proportion Z-tests. These tests were used to estimate *P*-values for the differences in each variant’s allele frequencies. The odds ratios were calculated to evaluate the strength of the association between allele frequencies in the two cohorts. For multiple comparisons, we applied the Benjamini-Hochberg procedure for false discovery rate (FDR) correction, considering results statistically significant at a threshold of *P* < 0.05. All statistical analyses were performed using the Python programming language, utilizing libraries such as pandas, scipy, and statsmodels.

### Functional consequences of rare and unreported variants

For the functional annotation of variants associated with TNFi response, we employed different machine learning tools including SIFT (61), POLYPHEN-2 (62), CADD (63), MutPred (64), M-CAP (45), DANN (65), VEP (66), dbNSFP (67), VEST3 (68), REVEAL (69), PROVEAN (70), Mutation Taster (71), DeepPVP (72), FATHMM (73), VariantRanker (74), PON-P2 (75), MetaSVM (76), MVP (77), and ANNOVAR (78).

### Cumulative risk probability

We calculated the Cumulative Allele Probability (CAP) metric, which incorporates both the count of functional variants and their allele frequencies for each gene, allowing us to estimate the probability that a population possesses at least one variant allele *a* from all observed alleles *A* in gene *g*. (79):


$$CAP(g)=1-\;\prod_{a\in A}{\left(1-AF(a)\right)}^{2}$$

In this study, CAP score was calculated for all the missense variants separately. Additionally, we calculated CAP score for the loss of function variants in TNFi response related genes.

### Molecular modeling and docking of the novel candidate variants

The crystal structures of TNF (PDB ID: 2AZ5) and TNFR1 (PDB ID: 2ZJC) were initially obtained for molecular modeling from the Protein Data Bank (PDB). The Swiss model server was used to build mutant models using the TNF crystal structure as a template, and the quality of the generated model was evaluated using the global model quality estimation (GMQE) score. In addition, AlphaFold was used to improve the accuracy and dependability of the protein structure predictions (80). The AlphaFold models were compared to the TNF crystal structure and mutant models created with the Swiss model server. To evaluate the stereochemical quality of the wild-type and mutant models, we used multiple programs, including ERRAT, Procheck and Verify3D (81). Finally, using PyMOL (v2.5) software, all of the created protein models, including those generated by AlphaFold, were analyzed (82). HADDOCK (v.2.4) (83) was used to perform protein-protein docking.

## Data Availability

The informed consent given by the study participants does not cover posting of participant level phenotype and genotype data of Qatar Biobank/Qatar Genome Project in public databases. However, access to the data can be obtained through an established ISO-certified process by submitting a project request at https://www.qphi.org.qa/research/how-to-apply which is subject to approval by the QBB IRB committee. Other relevant data are provided in the supplementary files.

## Pharmacogenomics of TNFi in Qatar (QPGx-TNFi) Consortium

College of Health & Life Sciences (CHLS), Hamad Bin Khalifa University, Doha, Qatar: Borbala Mifsud, Georges Nemer, Puthen Veettil Jithesh, Nady El Hajj, Ehsan Pourkarimi, Omar Albagha, Farah El Assadi, Zainab Jan, Dinesh Velayutham.

Rheumatology Department, Hamad Medical Corporation (HMC), Doha, Qatar: Samar Al Emadi, Karima Becetti, Mohammed Hammoudeh, Hala Albakheet, Nour Hamad, Sanaa Sharari.

Pediatric Rheumatology, Sidra Medicine, Doha, Qatar: Sharon Bout-Tabaku, Buthaina Al Adba, Mona El Chawli, Fathima Abubacker.

Pediatric Gastroenterology, Sidra Medicine, Doha, Qatar: Mamoun Elawad, Nazira Ibrahim.

## References

[1] Adelowo O Mody GM Tikly M Oyoo O Slimani S . Rheumatic diseases in Africa. Nat Rev Rheumatol. (2021) 17:363–74. doi: 10.1038/s41584-021-00603-4, PMID: 33850309 PMC8043097

[2] Angum F Khan T Kaler J Siddiqui L Hussain A . The Prevalence of Autoimmune Disorders in Women: A Narrative Review. Cureus. (2020) 12:e8094. doi: 10.7759/cureus.8094, PMID: 32542149 PMC7292717

[3] El-Tahan RR Ghoneim AM El-Mashad N . TNF-α gene polymorphisms and expression. SpringerPlus. (2016) 5:1508. doi: 10.1186/s40064-016-3197-y, PMID: 27652081 PMC5014780

[4] Conrad C Di Domizio J Mylonas A Belkhodja C Demaria O Navarini AA . TNF blockade induces a dysregulated type I interferon response without autoimmunity in paradoxical psoriasis. Nat Commun. (2018) 9:25. doi: 10.1038/s41467-017-02466-4, PMID: 29295985 PMC5750213

[5] van Loo G Bertrand MJM . Death by TNF: a road to inflammation. Nat Rev Immunol. (2023) 23:289–303. doi: 10.1038/s41577-022-00792-3, PMID: 36380021 PMC9665039

[6] Willrich MAV Murray DL Snyder MR . Tumor necrosis factor inhibitors: clinical utility in autoimmune diseases. Transl Res J Lab Clin Med. (2015) 165:270–82. doi: 10.1016/j.trsl.2014.09.006, PMID: 25305470

[7] Jung SM Kim W-U . Targeted Immunotherapy for Autoimmune Disease. Immune Netw. (2022) 22:e9. doi: 10.4110/in.2022.22.e9, PMID: 35291650 PMC8901705

[8] Nicolela Susanna F Pavesio C . A review of ocular adverse events of biological anti-TNF drugs. J Ophthalmic Inflamm Infect. (2020) 10:11. doi: 10.1186/s12348-020-00202-6, PMID: 32337619 PMC7184065

[9] Johnson KJ Sanchez HN Schoenbrunner N . Defining response to TNF-inhibitors in rheumatoid arthritis: the negative impact of anti-TNF cycling and the need for a personalized medicine approach to identify primary non-responders. Clin Rheumatol. (2019) 38:2967–76. doi: 10.1007/s10067-019-04684-1, PMID: 31520227

[10] Taylor PC Matucci Cerinic M Alten R Avouac J Westhovens R . Managing inadequate response to initial anti-TNF therapy in rheumatoid arthritis: optimising treatment outcomes. Ther Adv Musculoskelet Dis. (2022) 14:1759720X221114101. doi: 10.1177/1759720X221114101, PMID: 35991524 PMC9386864

[11] Rubbert-Roth A Szabó MZ Kedves M Nagy G Atzeni F Sarzi-Puttini P . Failure of anti-TNF treatment in patients with rheumatoid arthritis: The pros and cons of the early use of alternative biological agents. Autoimmun Rev. (2019) 18:102398. doi: 10.1016/j.autrev.2019.102398, PMID: 31639514

[12] Nielsen OH Ainsworth MA . Tumor Necrosis Factor Inhibitors for Inflammatory Bowel Disease. N Engl J Med. (2013) 369:754–62. doi: 10.1056/NEJMct1209614, PMID: 23964937

[13] Roda G Jharap B Neeraj N Colombel J-F . Loss of Response to Anti-TNFs: Definition, Epidemiology, and Management. Clin Transl Gastroenterol. (2016) 7:e135. doi: 10.1038/ctg.2015.63, PMID: 26741065 PMC4737871

[14] Jan Z El Assadi F Velayutham D Mifsud B Jithesh PV . Pharmacogenomics of TNF inhibitors. Front Immunol. (2025) 16:1521794. doi: 10.3389/fimmu.2025.1521794, PMID: 40469293 PMC12133927

[15] Sazonovs A Kennedy NA Moutsianas L Heap GA Rice DL Reppell M . HLA-DQA1*05 Carriage Associated With Development of Anti-Drug Antibodies to Infliximab and Adalimumab in Patients With Crohn’s Disease. Gastroenterology. (2020) 158:189–99. doi: 10.1053/j.gastro.2019.09.041, PMID: 31600487

[16] Wilson A Peel C Wang Q Pananos AD Kim RB . *HLADQA1*05* genotype predicts anti-drug antibody formation and loss of response during infliximab therapy for inflammatory bowel disease. Aliment Pharmacol Ther. (2020) 51:356–63. doi: 10.1111/apt.15563, PMID: 31650614

[17] Massey J Plant D Hyrich K Morgan AW Wilson AG Spiliopoulou A . Genome-wide association study of response to tumour necrosis factor inhibitor therapy in rheumatoid arthritis. Pharmacogenomics J. (2018) 18:657–64. doi: 10.1038/s41397-018-0040-6, PMID: 30166627 PMC6150911

[18] Padula MC Leccese P Lascaro N Radice RP Limongi AR Sorrento GG . Correlation of Tumor Necrosis Factor-α –308G>A Polymorphism with Susceptibility, Clinical Manifestations, and Severity in Behçet Syndrome: Evidences from an Italian Genetic Case–Control Study. DNA Cell Biol. (2020) 39:1104–10. doi: 10.1089/dna.2020.5361, PMID: 32352842

[19] Padula MC Padula AA D’Angelo S Lascaro N Radice RP Martelli G . TNFα rs1800629 Polymorphism and Response to Anti-TNFα Treatment in Behçet Syndrome: Data from an Italian Cohort Study. J Pers Med. (2023) 13:1347. doi: 10.3390/jpm13091347, PMID: 37763115 PMC10532840

[20] Badsha H Kong KO Tak PP . Rheumatoid arthritis in the United Arab Emirates. Clin Rheumatol. (2008) 27:739–42. doi: 10.1007/s10067-007-0782-z, PMID: 17973153

[21] Saxena R Plenge RM Bjonnes AC Dashti HS Okada Y Gad El Haq W . A Multinational Arab Genome-Wide Association Study Identifies New Genetic Associations for Rheumatoid Arthritis. Arthritis Rheumatol. (2017) 69:976–85. doi: 10.1002/art.40051, PMID: 28118524

[22] Hammoudeh M Elsayed E Al-Kaabi S Sharma M Elbadri M Chandra P . Rheumatic manifestations of inflammatory bowel diseases: A study from the Middle East. J Int Med Res. (2018) 46:3837–47. doi: 10.1177/0300060518781404, PMID: 29961404 PMC6136032

[23] Alam F Hammoudeh M Chandra P Al Emadi S . THU0091 Treatment Pattern and Disease Activity Scores in Rheumatoid Arthritis Patients; Data Taken from Qatar Rheumatoid Arthritis Registry. Ann Rheumatol Dis. (2016) 75:212.1–212. doi: 10.1136/annrheumdis-2016-eular.1203

[24] Bek S Bojesen AB Nielsen JV Sode J Bank S Vogel U . Systematic review and meta-analysis: pharmacogenetics of anti-TNF treatment response in rheumatoid arthritis. Pharmacogenomics J. (2017) 17:403–11. doi: 10.1038/tpj.2017.26, PMID: 28607508 PMC5637244

[25] Padyukov L Lampa J Heimbürger M Ernestam S Cederholm T Lundkvist I . Genetic markers for the efficacy of tumour necrosis factor blocking therapy in rheumatoid arthritis. Ann Rheumatol Dis. (2003) 62:526–9. doi: 10.1136/ard.62.6.526, PMID: 12759288 PMC1754569

[26] Kang CP Lee KW Yoo DH Kang C Bae SC . The influence of a polymorphism at position -857 of the tumour necrosis factor alpha gene on clinical response to etanercept therapy in rheumatoid arthritis. Rheumatol Oxf Engl. (2005) 44:547–52. doi: 10.1093/rheumatology/keh550, PMID: 15695296

[27] Chen Y-Y . Correlations of CYP2C9*3/CYP2D6*10/CYP3A5*3 gene polymorphisms with efficacy of etanercept treatment for patients with ankylosing spondylitis: A case-control study. Med (Baltimore). (2017) 96:e5993. doi: 10.1097/MD.0000000000005993, PMID: 28248857 PMC5340430

[28] Acosta-Colman I Palau N Tornero J Fernández-Nebro A Blanco F González-Alvaro I . GWAS replication study confirms the association of PDE3A-SLCO1C1 with anti-TNF therapy response in rheumatoid arthritis. Pharmacogenomics. (2013) 14:727–34. doi: 10.2217/pgs.13.60, PMID: 23651021

[29] Rooryck C Barnetche T Richez C Laleye A Arveiler B Schaeverbeke T . Influence of FCGR3A-V212F and TNFRSF1B-M196R genotypes in patients with rheumatoid arthritis treated with infliximab therapy. Clin Exp Rheumatol. (2008) 26:340–2., PMID: 18565259

[30] Ovejero-Benito MC Prieto-Pérez R Llamas-Velasco M Belmonte C Cabaleiro T Román M . Polymorphisms associated with etanercept response in moderate-to-severe plaque psoriasis. Pharmacogenomics. (2017) 18:631–8. doi: 10.2217/pgs-2017-0014, PMID: 28470127

[31] Canet LM Filipescu I Cáliz R Lupiañez CB Canhão H Escudero A . Genetic variants within the TNFRSF1B gene and susceptibility to rheumatoid arthritis and response to anti-TNF drugs: a multicenter study. Pharmacogenet Genomics. (2015) 25:323–33. doi: 10.1097/FPC.0000000000000140, PMID: 25850964

[32] Stavrou EF Chatzopoulou F Antonatos C Pappa P Makridou E Oikonomou K . Pharmacogenetic analysis of canonical versus noncanonical pathway of NF-kB in Crohn’s disease patients under anti-tumor necrosis factor-α treatment. Pharmacogenet Genomics. (2022) 32:235–41. doi: 10.1097/FPC.0000000000000471, PMID: 35852914

[33] Liu C Batliwalla F Li W Lee A Roubenoff R Beckman E . Genome-Wide Association Scan Identifies Candidate Polymorphisms Associated with Differential Response to Anti-TNF Treatment in Rheumatoid Arthritis. Mol Med. (2008) 14:575–81. doi: 10.2119/2008-00056.Liu, PMID: 18615156 PMC2276142

[34] Antonatos C Stavrou EF Evangelou E Vasilopoulos Y . Exploring Pharmacogenetic Variants for Predicting Response to Anti-TNF Therapy in Autoimmune Diseases: a Meta-Analysis. Pharmacogenomics. (2021) 22:435–45. doi: 10.2217/pgs-2021-0019, PMID: 33887993

[35] Lacruz-Guzmán D Torres-Moreno D Pedrero F Romero-Cara P García-Tercero I Trujillo-Santos J . Influence of polymorphisms and TNF and IL1β serum concentration on the infliximab response in Crohn’s disease and ulcerative colitis. Eur J Clin Pharmacol. (2013) 69:431–8. doi: 10.1007/s00228-012-1389-0, PMID: 22960943

[36] Repnik K Koder S Skok P Ferkolj I Potočnik U . Transferrin Level Before Treatment and Genetic Polymorphism in HFE Gene as Predictive Markers for Response to Adalimumab in Crohn’s Disease Patients. Biochem Genet. (2016) 54:476–86. doi: 10.1007/s10528-016-9734-0, PMID: 27115882

[37] Goldstein DB Allen A Keebler J Margulies EH Petrou S Petrovski S . Sequencing studies in human genetics: design and interpretation. Nat Rev Genet. (2013) 14:460–70. doi: 10.1038/nrg3455, PMID: 23752795 PMC4117319

[38] Nelson MR Wegmann D Ehm MG Kessner D St Jean P Verzilli C . An abundance of rare functional variants in 202 drug target genes sequenced in 14,002 people. Science. (2012) 337:100–4. doi: 10.1126/science.1217876, PMID: 22604722 PMC4319976

[39] Wright GEB Carleton B Hayden MR Ross CJD . The global spectrum of protein-coding pharmacogenomic diversity. Pharmacogenomics J. (2018) 18:187–95. doi: 10.1038/tpj.2016.77, PMID: 27779249 PMC5817389

[40] Auer PL Lettre G . Rare variant association studies: considerations, challenges and opportunities. Genome Med. (2015) 7:16. doi: 10.1186/s13073-015-0138-2, PMID: 25709717 PMC4337325

[41] Bluett J Ibrahim I Plant D Hyrich KL Morgan AW Wilson AG . Association of a complement receptor 1 gene variant with baseline erythrocyte sedimentation rate levels in patients starting anti-TNF therapy in a UK rheumatoid arthritis cohort: results from the Biologics in Rheumatoid Arthritis Genetics and Genomics Study Syndicate cohort. Pharmacogenomics J. (2014) 14:171–5. doi: 10.1038/tpj.2013.26, PMID: 23856853 PMC3965567

[42] Kullo IJ Ding K Shameer K McCarty CA Jarvik GP Denny JC . Complement Receptor 1 Gene Variants Are Associated with Erythrocyte Sedimentation Rate. Am J Hum Genet. (2011) 89:131–8. doi: 10.1016/j.ajhg.2011.05.019, PMID: 21700265 PMC3135803

[43] Chadwick A Frazier A Khan TW Young E . Understanding the Psychological, Physiological, and Genetic Factors Affecting Precision Pain Medicine: A Narrative Review. J Pain Res. (2021) 14:3145–61. doi: 10.2147/JPR.S320863, PMID: 34675643 PMC8517910

[44] Ritchie GR Flicek P . Computational approaches to interpreting genomic sequence variation. Genome Med. (2014) 6:87. doi: 10.1186/s13073-014-0087-1, PMID: 25473426 PMC4254438

[45] Jagadeesh KA Wenger AM Berger MJ Guturu H Stenson PD Cooper DN . M-CAP eliminates a majority of variants of uncertain significance in clinical exomes at high sensitivity. Nat Genet. (2016) 48:1581–6. doi: 10.1038/ng.3703, PMID: 27776117

[46] Ma Y Mu J Gou X Wu X . Precision medication based on the evaluation of drug metabolizing enzyme and transporter functions. Precis Clin Med. (2025) 8:pbaf004. doi: 10.1093/pcmedi/pbaf004, PMID: 40110576 PMC11920622

[47] Mbarek H Gandhi GD Selvaraj S Al-Muftah W Badji R Al-Sarraj Y . Qatar Genome: Insights on Genomics from the Middle East (preprint). Genet Genomic Med. (2021) 43(4):499–510. doi: 10.1101/2021.09.19.21263548, PMID: 35112413

[48] Jithesh PV Abuhaliqa M Syed N Ahmed I El Anbari M Bastaki K . A population study of clinically actionable genetic variation affecting drug response from the Middle East. NPJ Genomic Med. (2022) 7:10. doi: 10.1038/s41525-022-00281-5, PMID: 35169154 PMC8847489

[49] Li H Durbin R . Fast and accurate long-read alignment with Burrows–Wheeler transform. Bioinformatics. (2010) 26:589–95. doi: 10.1093/bioinformatics/btp698, PMID: 20080505 PMC2828108

[50] Freed D Aldana R Weber JA Edwards JS . The Sentieon Genomics Tools - A fast and accurate solution to variant calling from next-generation sequence data. (2017). doi: 10.1101/115717

[51] Gong L Whirl-Carrillo M Klein TE . PharmGKB, an Integrated Resource of Pharmacogenomic Knowledge. Curr Protoc. (2021) 1:e226. doi: 10.1002/cpz1.226, PMID: 34387941 PMC8650697

[52] Al-Sofi RF Bergmann MS Nielsen CH Andersen V Skov L Loft N . The Association between Genetics and Response to Treatment with Biologics in Patients with Psoriasis, Psoriatic Arthritis, Rheumatoid Arthritis, and Inflammatory Bowel Diseases: A Systematic Review and Meta-Analysis. Int J Mol Sci. (2024) 25:5793. doi: 10.3390/ijms25115793, PMID: 38891983 PMC11171831

[53] Genovese G Rockweiler NB Gorman BR Bigdeli TB Pato MT Pato CN . BCFtools/liftover: an accurate and comprehensive tool to convert genetic variants across genome assemblies. Bioinformatics. (2024) 40:btae038. doi: 10.1093/bioinformatics/btae038, PMID: 38261650 PMC10832354

[54] Gudmundsson S Singer-Berk M Watts NA Phu W Goodrich JK Solomonson M . Variant interpretation using population databases: Lessons from gnomAD. Hum Mutat. (2021) 43(8):1012–30. doi: 10.1002/humu.24309, PMID: 34859531 PMC9160216

[55] Jarvik GP Browning BL . Consideration of Cosegregation in the Pathogenicity Classification of Genomic Variants. Am J Hum Genet. (2016) 98:1077–81. doi: 10.1016/j.ajhg.2016.04.003, PMID: 27236918 PMC4908147

[56] González-Pérez A López-Bigas N . Improving the assessment of the outcome of nonsynonymous SNVs with a consensus deleteriousness score, Condel. Am J Hum Genet. (2011) 88:440–9. doi: 10.1016/j.ajhg.2011.03.004, PMID: 21457909 PMC3071923

[57] McLaren W Gil L Hunt SE Riat HS Ritchie GRS Thormann A . The Ensembl Variant Effect Predictor. Genome Biol. (2016) 17:122. doi: 10.1186/s13059-016-0974-4, PMID: 27268795 PMC4893825

[58] Karczewski KJ Francioli LC Tiao G Cummings BB Alföldi J Wang Q . The mutational constraint spectrum quantified from variation in 141,456 humans. Nature. (2020) 581:434–43. doi: 10.1038/s41586-020-2308-7, PMID: 32461654 PMC7334197

[59] Hari A Zhou Q Gonzaludo N Harting J Scott SA Sahinalp SC . Aldy 4: An efficient genotyper and star-allele caller for pharmacogenomics. Genome Research. (2022) 33:61–70. doi: 10.1101/2022.08.11.503701, PMID: 36657977 PMC9977157

[60] Purcell S Neale B Todd-Brown K Thomas L Ferreira MAR Bender D . PLINK: A Tool Set for Whole-Genome Association and Population-Based Linkage Analyses. Am J Hum Genet. (2007) 81:559–75. doi: 10.1086/519795, PMID: 17701901 PMC1950838

[61] Vaser R Adusumalli S Leng SN Sikic M Ng PC . SIFT missense predictions for genomes. Nat Protoc. (2016) 11:1–9. doi: 10.1038/nprot.2015.123, PMID: 26633127

[62] Adzhubei I Jordan DM Sunyaev SR . Predicting functional effect of human missense mutations using PolyPhen-2. Curr Protoc Hum Genet. (2013) 76:7–20. doi: 10.1002/0471142905.hg0720s76, PMID: 23315928 PMC4480630

[63] Rentzsch P Witten D Cooper GM Shendure J Kircher M . CADD: predicting the deleteriousness of variants throughout the human genome. Nucleic Acids Res. (2019) 47:D886–94. doi: 10.1093/nar/gky1016, PMID: 30371827 PMC6323892

[64] Mort M Sterne-Weiler T Li B Ball EV Cooper DN Radivojac P . MutPred Splice: machine learning-based prediction of exonic variants that disrupt splicing. Genome Biol. (2014) 15:R19. doi: 10.1186/gb-2014-15-1-r19, PMID: 24451234 PMC4054890

[65] Quang D Chen Y Xie X . DANN: a deep learning approach for annotating the pathogenicity of genetic variants. Bioinforma Oxf Engl. (2015) 31:761–3. doi: 10.1093/bioinformatics/btu703, PMID: 25338716 PMC4341060

[66] Hunt SE Moore B Amode RM Armean IM Lemos D Mushtaq A . Annotating and prioritizing genomic variants using the Ensembl Variant Effect Predictor-A tutorial. Hum Mutat. (2021) 43:986–97. doi: 10.22541/au.162460842.27880071/v1, PMID: 34816521 PMC7613081

[67] Liu X Jian X Boerwinkle E . dbNSFP v2.0: a database of human non-synonymous SNVs and their functional predictions and annotations. Hum Mutat. (2013) 34:E2393–2402. doi: 10.1002/humu.22376, PMID: 23843252 PMC4109890

[68] Carter H Douville C Stenson PD Cooper DN Karchin R . Identifying Mendelian disease genes with the variant effect scoring tool. BMC Genomics. (2013) 14 Suppl 3:S3. doi: 10.1186/1471-2164-14-S3-S3, PMID: 23819870 PMC3665549

[69] Ioannidis NM Rothstein JH Pejaver V Middha S McDonnell SK Baheti S . REVEL: An Ensemble Method for Predicting the Pathogenicity of Rare Missense Variants. Am J Hum Genet. (2016) 99:877–85. doi: 10.1016/j.ajhg.2016.08.016, PMID: 27666373 PMC5065685

[70] Choi Y Chan AP . PROVEAN web server: a tool to predict the functional effect of amino acid substitutions and indels. Bioinforma Oxf Engl. (2015) 31:2745–7. doi: 10.1093/bioinformatics/btv195, PMID: 25851949 PMC4528627

[71] Schwarz JM Cooper DN Schuelke M Seelow D . MutationTaster2: mutation prediction for the deep-sequencing age. Nat Methods. (2014) 11:361–2. doi: 10.1038/nmeth.2890, PMID: 24681721

[72] Boudellioua I Kulmanov M Schofield PN Gkoutos GV Hoehndorf R . DeepPVP: phenotype-based prioritization of causative variants using deep learning. BMC Bioinf. (2019) 20:65. doi: 10.1186/s12859-019-2633-8, PMID: 30727941 PMC6364462

[73] Shihab HA Gough J Cooper DN Stenson PD Barker GLA Edwards KJ . Predicting the functional, molecular, and phenotypic consequences of amino acid substitutions using hidden Markov models. Hum Mutat. (2013) 34:57–65. doi: 10.1002/humu.22225, PMID: 23033316 PMC3558800

[74] Alexander J Mantzaris D Georgitsi M Drineas P Paschou P . Variant Ranker: a web-tool to rank genomic data according to functional significance. BMC Bioinf. (2017) 18:341. doi: 10.1186/s12859-017-1752-3, PMID: 28716001 PMC5514526

[75] Niroula A Urolagin S Vihinen M . PON-P2: prediction method for fast and reliable identification of harmful variants. PloS One. (2015) 10:e0117380. doi: 10.1371/journal.pone.0117380, PMID: 25647319 PMC4315405

[76] Zaucha J Heinzinger M Tarnovskaya S Rost B Frishman D . Family-specific analysis of variant pathogenicity prediction tools. NAR Genomics Bioinforma. (2020) 2:lqaa014. doi: 10.1093/nargab/lqaa014, PMID: 33575576 PMC7671395

[77] Qi H Zhang H Zhao Y Chen C Long JJ Chung WK . MVP predicts the pathogenicity of missense variants by deep learning. Nat Commun. (2021) 12:510. doi: 10.1038/s41467-020-20847-0, PMID: 33479230 PMC7820281

[78] Wang K Li M Hakonarson H . ANNOVAR: functional annotation of genetic variants from high-throughput sequencing data. Nucleic Acids Res. (2010) 38:e164. doi: 10.1093/nar/gkq603, PMID: 20601685 PMC2938201

[79] Schärfe CPI Tremmel R Schwab M Kohlbacher O Marks DS . Genetic variation in human drug-related genes. Genome Med. (2017) 9:117. doi: 10.1186/s13073-017-0502-5, PMID: 29273096 PMC5740940

[80] Jumper J Evans R Pritzel A Green T Figurnov M Ronneberger O . Highly accurate protein structure prediction with AlphaFold. Nature. (2021) 596:583–9. doi: 10.1038/s41586-021-03819-2, PMID: 34265844 PMC8371605

[81] Laskowski RA Rullmannn JA MacArthur MW Kaptein R Thornton JM . AQUA and PROCHECK-NMR: programs for checking the quality of protein structures solved by NMR. J Biomol NMR. (1996) 8:477–86. doi: 10.1007/BF00228148, PMID: 9008363

[82] Schrödinger L . The PyMOL molecular graphics system. Version, 1, 8.

[83] Dominguez C Boelens R Bonvin AMJJ . HADDOCK: a protein-protein docking approach based on biochemical or biophysical information. J Am Chem Soc. (2003) 125:1731–7. doi: 10.1021/ja026939x, PMID: 12580598

