# Supplementary material for: Pharmacogenomic landscape of TNF inhibitors in the Middle Eastern Qatari population
Source: Front Immunol. 2025 Nov 19;16:1674889. doi: 10.3389/fimmu.2025.1674889 (PMC12672520; doi:10.3389/fimmu.2025.1674889)
Supplement: Supplementary file 1 [file Table1.docx]

**Supplementary Figures**

**
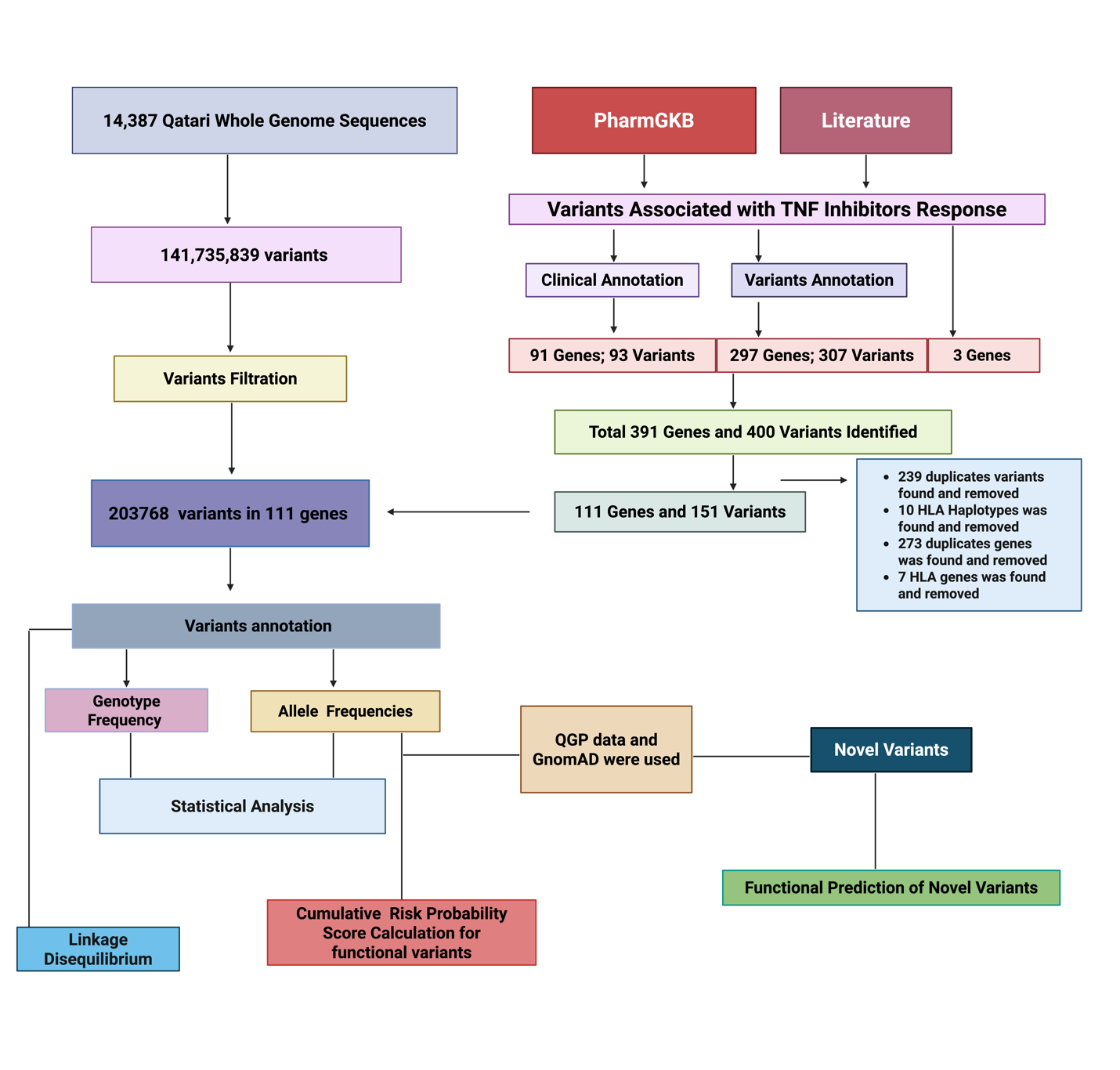
**

**Fig. S1.** Workflow used for the identification of variants associated with response to TNF inhibitors.


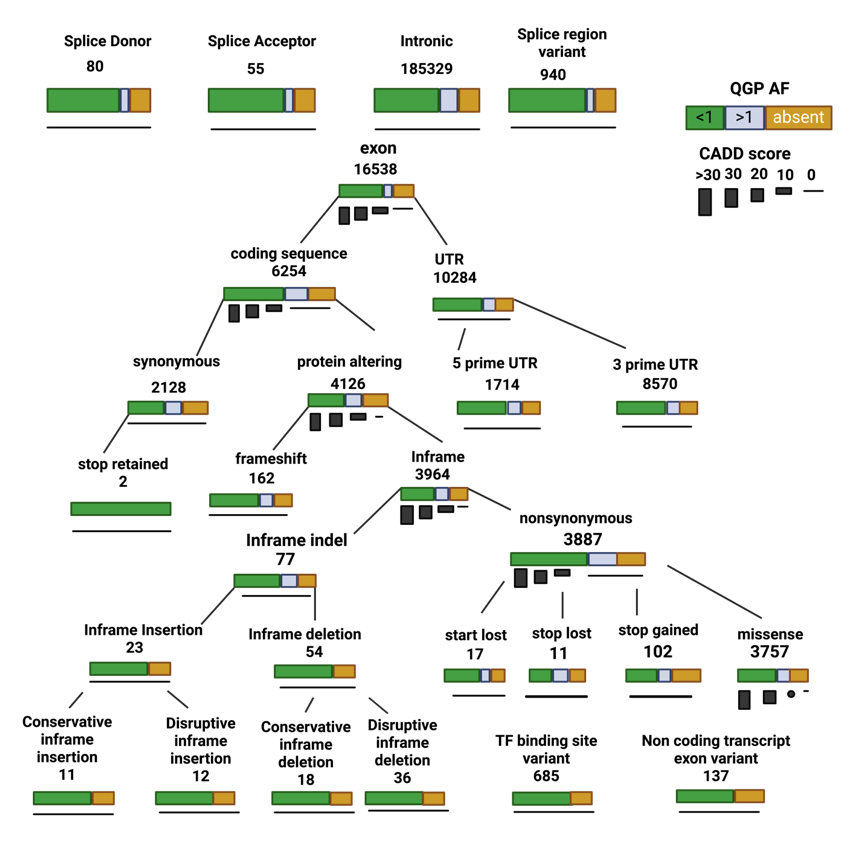


**Fig. S2.** Comparison of variants in TNF inhibitor (TNFi) response-associated genes in the Qatar Genome Project (QGP) data versus gnomAD. A total of 203,768 variants were analyzed to calculate these statistics. Yellow highlights variants present in gnomAD but absent in QGP, while grey indicates variants with allele frequencies greater than 1%. Green represents variants with allele frequencies less than 1%. Black boxes denote CADD scores. These genes have been previously linked to TNFi response in other populations; however, their association with TNFi response in the Qatari population has yet to be determined.


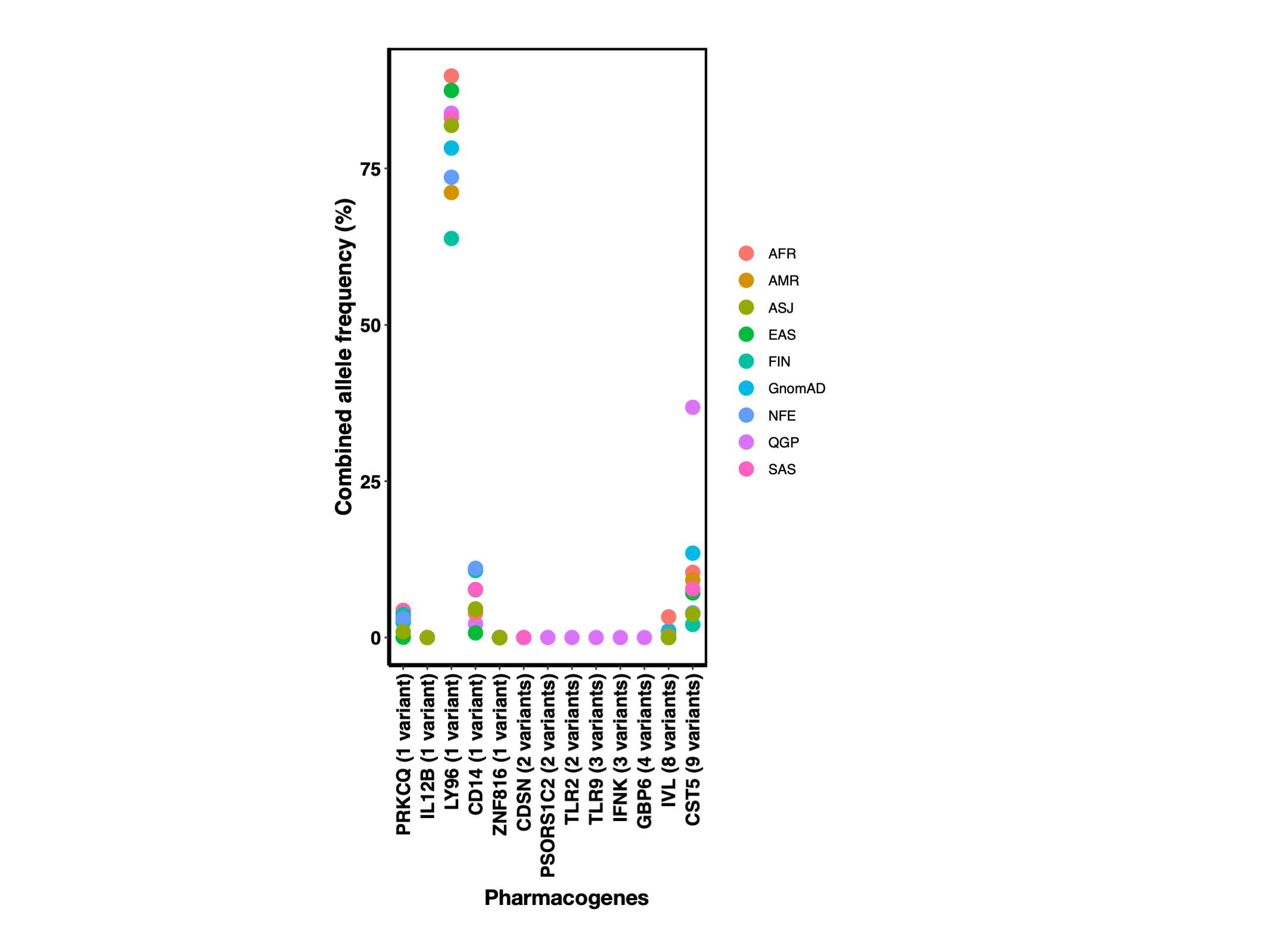


**Fig. S3.** Combined allele frequency for Loss of Function variants in the Qatari population compared with other world populations.


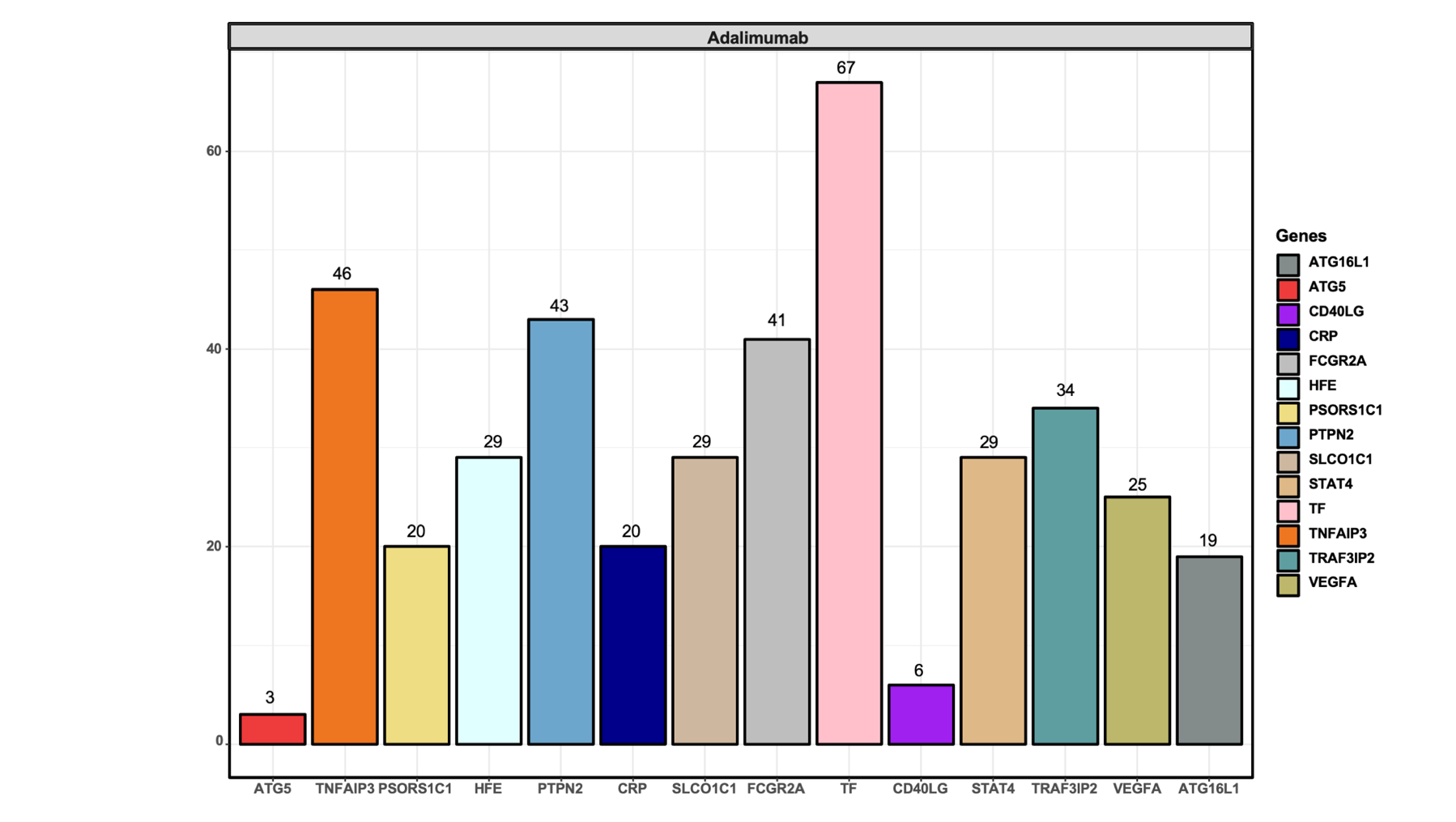


**Fig. S4.** Summary of variants associated with Adalimumab response identified in other populations and their prevalence in the Qatari population. The figure illustrates the number of functional genetic variants per participant for key genes associated with the response to Adalimumab.


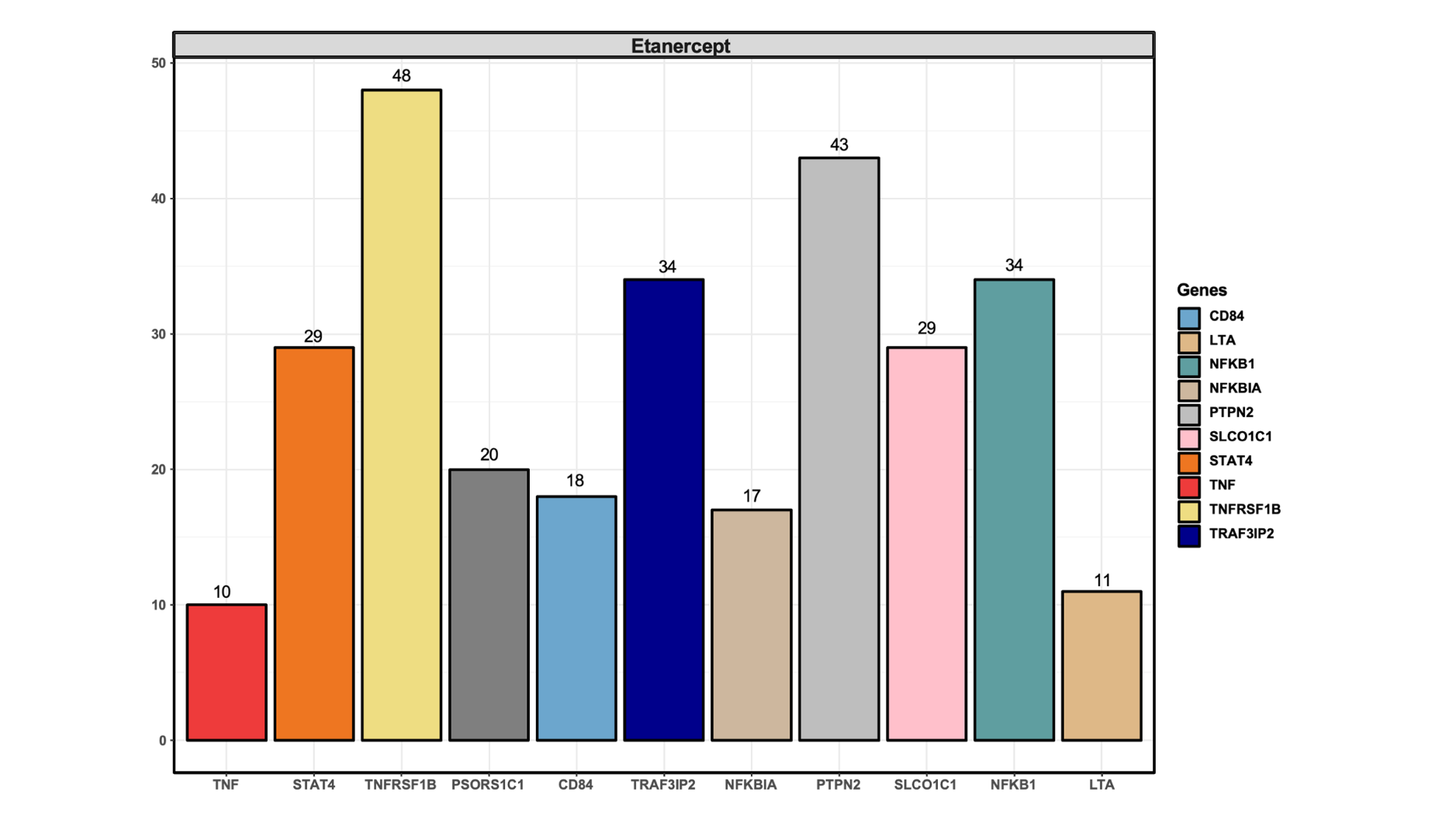


**Fig. S5.** Summary of variants associated with Etanercept response identified in other populations and their prevalence in the Qatari population. The figure illustrates the number of functional genetic variants per participant for key genes associated with the response to Etanercept.


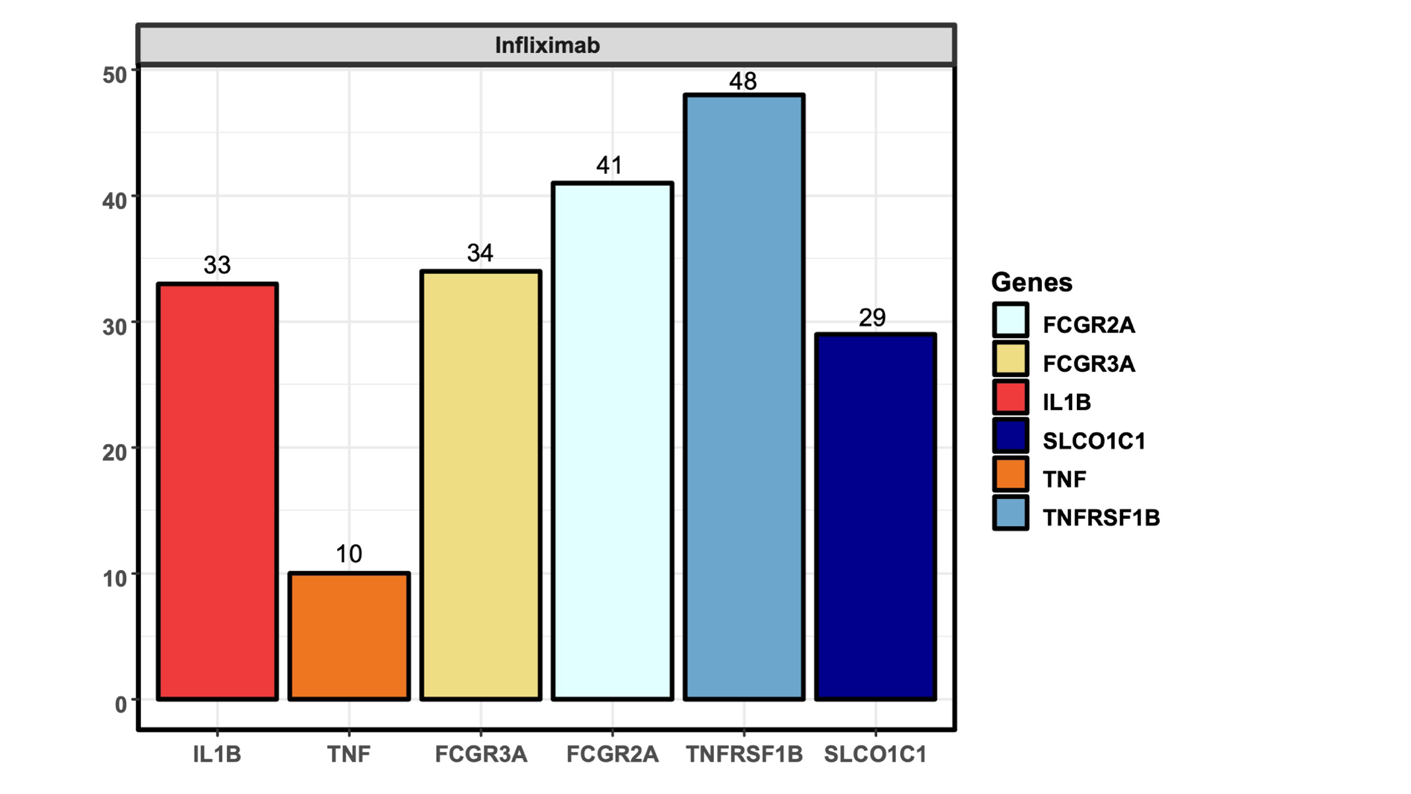


**Fig. S6.** Summary of variants associated with Infliximab response identified in other populations and their prevalence in the Qatari population. The figure illustrates the number of functional genetic variants per participant for key genes associated with the response to Infliximab.
